# Impact of Medical Conditions and Area Deprivation on Fundraising Success in Online Crowdfunding: Cross-Sectional Study

**DOI:** 10.2196/72475

**Published:** 2025-07-29

**Authors:** Steven S Doerstling, Matthew M Engelhard, Dennis Akrobetu, Caroline E Sloan, Ada Campagna, Thuy-Vi Nguyen, Farrah Madanay, Felicia Chen, Peter A Ubel

**Affiliations:** 1 Department of Medicine, School of Medicine, Stanford University, 300 Pasteur Dr, Stanford, CA, 94305, United States, 1 6507252504; 2 Department of Biostatistics & Bioinformatics, Duke University, Durham, NC, United States; 3 Department of Ophthalmology, Massachusetts Eye and Ear, Harvard Medical School, Cambridge, MA, United States; 4 Department of Medicine, School of Medicine, Duke University, Durham, NC, United States; 5 Department of Population Health Sciences, School of Medicine, Duke University, Durham, NC, United States; 6 Duke-Margolis Center for Health Policy, Duke University, Durham, NC, United States; 7 Department of Sociology, University of California, Los Angeles, Los Angeles, CA, United States; 8 Miller School of Medicine, University of Miami, Coral Gables, FL, United States; 9 Center for Bioethics and Social Sciences in Medicine, Medical School, University of Michigan–Ann Arbor, Ann Arbor, MI, United States; 10 GrantScout, San Francisco, CA, United States; 11 Sanford School of Public Policy, Duke University, Durham, NC, United States; 12 Fuqua School of Business, Duke University, Durham, NC, United States

**Keywords:** crowdfunding, health care costs, health disparities

## Abstract

**Background:**

Web-based crowdfunding is commonly used to defray medical expenses, but it is not fully known which factors determine fundraising success. Previous studies have usually focused on a single disease category at a time or a small number of mutually exclusive diseases, even though a given campaign may seek funding for multiple conditions. In addition, differences in fundraising exist according to socioeconomic status, but whether this association applies across different diseases is unclear. Thus, questions remain about how certain medical conditions and the socioeconomic context of a campaign’s location interact to influence fundraising success.

**Objective:**

This study aimed to determine the impact of specific medical conditions on crowdfunding success and to evaluate if the socioeconomic environment of a campaign’s location has a distinct effect on earnings. Last, we sought to understand the effect of these features on donation behavior in terms of number of donations and donation amount.

**Methods:**

Web scraping was used to collect medical crowdfunding campaigns on GoFundMe that were based in the United States and created between 2010 and 2020. Using a previously validated disease identification algorithm based on natural language processing, we identified the presence or absence of 11 broad disease categories in each campaign description. An Area Deprivation Index was calculated to represent a composite view of the socioeconomic status of each campaign’s county of origin. Generalized linear models were constructed to estimate the impact of mentioning specific disease categories and the campaign’s area deprivation on the amount of money raised.

**Results:**

This study analyzed 89,645 crowdfunding campaigns. We identified at least one medical condition in 82.6% (n=74,016) of campaigns. A quarter of campaigns (n=25,026, 27.9%) mentioned more than one disease category. Neoplasms were the most common condition among medical crowdfunding campaigns by a large margin (n=38,221, 43.7% of campaigns), followed by injuries and external causes (n=18,087, 20.7% of campaigns). In multivariable analysis, mentioning neoplasms, injuries and external causes, respiratory system diseases, nervous system diseases, or infections in the campaign was associated with higher total fundraising amounts. On the other hand, mentioning genitourinary, mental health, or endocrine diseases was associated with lower total fundraising amounts. Campaigns originating from less-deprived counties raised more money than those from more-deprived counties, and this effect was independent of the diseases mentioned in the campaign. The success of campaigns for higher-earning conditions and from less-deprived areas was typically due to a larger number of donations, rather than a higher donation amount.

**Conclusions:**

The medical conditions mentioned in crowdfunding campaigns matter for the fundraising success of the campaign. Importantly, certain diseases tended to receive lower total fundraising amounts. Regardless of the specific diseases mentioned in the campaign, the socioeconomic backdrop of a campaign’s location had a significant impact on fundraising.

## Introduction

In the United States, many people have difficulty paying medical bills [1-3], and health care costs contribute to personal bankruptcy [4]. In this context, a growing number of patients use web-based crowdfunding to raise money for medical care. GoFundMe (GFM) is a popular crowdfunding platform [5,6], and nearly one-third of campaigns on GFM are for medical expenses [7]. The financial support made possible through crowdfunding is significant: one report calculated that more than US $3.6 billion has been raised by medical crowdfunding campaigns on GFM as of April 2019 [8].

Insufficient health insurance coverage leading to an out-of-pocket cost burden is frequently cited as a reason for seeking crowdfunding support [9,10]. Early research on medical crowdfunding focused on campaigns raising money for unapproved, scientifically unsupported, and potentially dangerous treatments that were not approved by health insurance programs [11-13]. However, further work has shown that web-based crowdfunding is a popular funding mechanism for conventional health care services and expenses indirectly related to medical care, such as housing and lost income [14,15].

Out-of-pocket costs vary across medical conditions [16], and previous work has sought to characterize which illnesses are most commonly described in medical crowdfunding campaigns. Special attention has been paid to conditions that are both common and associated with high out-of-pocket costs, including cancer [9,15,17-22], traumatic injuries [8,23], neurologic conditions [8,23-25], cardiovascular disease [8,23,26], and many others [27-30]. Despite the breadth of previous work, most studies have focused on either a single disease at a time or have categorized campaigns into a small number of mutually exclusive groups, even though patients with multiple medical conditions are especially prone to financial distress [31]. Open questions remain about the prevalence of multimorbidity in medical crowdfunding and the impact of other less-studied conditions, such as mental health disorders, on fundraising outcomes.

Fundraising success relies on a campaign’s exposure to potential donors, the likelihood of potential donors donating, and the value of individual contributions. Some donors may be more likely to contribute to campaigns that mention specific diseases [22]. It is also important to consider the socioeconomic context of a crowdfunding campaign in order to understand who is likely to earn financial support from web-based communities. Out-of-pocket cost burden disproportionately affects socioeconomically disadvantaged populations [32], but these same populations may also face barriers to seeking crowdfunding support in the first place, such as lack of internet access or simply being unfamiliar with web-based crowdfunding options [33]. Furthermore, socioeconomically deprived communities may have a smaller network of potential donors, and potential donors may make smaller contributions [34]. Previous work has shown that campaigns originating from socioeconomically deprived areas generally earn less [17,35], but it is unclear if this association applies across a broad range of disease categories.

With these remaining questions in mind, we designed a study to systematically analyze more disease categories than previous work in a large sample of medical crowdfunding campaigns and integrate the analysis with socioeconomic indicators linked to the campaigns’ location. We hypothesized that campaigns would frequently mention more than one disease category and that the association between certain diseases and campaign earnings would vary across disease categories. In addition, we hypothesized that socioeconomically deprived areas would be underrepresented in web-based crowdfunding and would be a significant determinant of fundraising success, regardless of the diseases mentioned in the campaign. Last, we analyzed the impact of medical conditions and area deprivation on the number and amount of individual donations, in an effort to understand how these features might underlie fundraising behavior.

## Methods

### Ethical Considerations

This study was approved by the Duke University institutional review board. The study was exempt as it was classified as a secondary analysis of existing data. The data were publicly available and were collected in accordance with the principles of fair use. Therefore, no consent was needed to proceed with the study. This manuscript follows the STROBE (Strengthening the Reporting of Observational Studies in Epidemiology) reporting guidelines for cross-sectional studies [36].

### Crowdfunding Data Collection

We wrote a web scraping program to collect data from a random sample of medical crowdfunding campaigns hosted by GFM. Eligible campaigns were those self-categorized as “Medical, Illness & Healing” and located in the United States. We only selected campaigns from this category to ensure that medical fundraising was the primary intent of the campaign because our disease identification algorithm (discussed below) is unable to distinguish between incidental and deliberate descriptions of medical problems. Per guidance from our institutional review board, we collected data from no more than 5% of campaigns available on the GFM sitemap, resulting in a maximum sample size of 100,000. We randomized the order of campaigns and scraped data until we collected approximately 100,000 campaigns. Web scraping was completed in August 2020. Variables that were collected include campaign title and description, amount of money sought and raised, zip code associated with the campaign, and date of creation. The language of each campaign description was determined using an automated language detector [37]. All data collected from GFM were publicly available and aggregated for research purposes in accordance with fair use. A discussion on the possibility of bias in our sample is available in Texts S1 and S2 and Figure S1 in Multimedia Appendix 1.

### Exclusion Criteria

Of the 99,943 crowdfunding campaigns collected by web scraping, we excluded campaigns that were duplicated (n=6981), had missing or unmappable zip codes (n=421), were located outside of the United States (n=234), had missing census data for the assigned county (n=6), were determined to be in a non-English language (n=2561), or had a currency code other than US dollars (n=25). To avoid campaigns seeking to raise money for research rather than medical expenses, we excluded campaigns with the word “research” in the campaign title (n=70).

### Disease Categorization Algorithm

We previously designed and validated an algorithm to identify the presence of broad disease categories in medical crowdfunding campaign descriptions [38]. Briefly, we used the 2021 Clinical Classifications Software Refined for *ICD-10-CM* (*International Classification of Diseases, Tenth Revision, Clinical Modification*) Diagnoses to establish 11 disease categories by which to summarize medical diagnoses. We used a pretrained named entity recognition model to identify segments of text in the campaign description that were predicted to represent medical diagnoses. Next, we used a pretrained entity resolution model to convert each segment of the text identified as a medical diagnosis into the best matching *ICD-10-CM* diagnosis code. Last, each *ICD-10-CM* code was matched to its corresponding disease, and each disease category was summarized as present or absent for every campaign. In parallel, the algorithm used a targeted keyword search for common treatments and procedures that indicate the presence of a certain disease category (eg, “heart transplant” suggesting the presence of cardiovascular disease) that appeared during our team’s exploratory reading. The algorithm was shown to have high precision, recall, and accuracy across disease categories when compared with manually-coded crowdfunding campaigns.

In certain cases, our algorithm did not identify any disease category in the campaign description. We reviewed a random sample of 100 of these campaigns and found that 75% either did not mention a medical diagnosis or conveyed a medical condition in vague terms; the remaining campaigns had various abnormalities such as excessive punctuation or nonspecific abbreviations (eg, “MS” for multiple sclerosis) in an atypical context. Complete classification performance metrics of the algorithm and discussion of the structure of the crowdfunding text are available in previous work [38].

### US Census Data and Area Deprivation Index

We performed a principal components analysis on county-level socioeconomic variables from the American Community Survey (ACS) to calculate an Area Deprivation Index (ADI) similar to previous work. The US Census Bureau’s API was used to collect county-level data from the 2019 ACS. The final ADI included unemployment rate, poverty rate, percent uninsured, percent who completed high school, percent with internet access, rate of single-parent households, and percentage of households with annual income <US $35,000. Weighted factor loadings for these variables were summed and standardized to generate the ADI value. Counties were then split into quartiles based on the ADI value (n=785 or 786 counties in each quartile, quartile 4=most deprived). Additional details on the principal components analysis and factor loading are available in Text S3 and Table S1 in Multimedia Appendix 1.

To link census data to crowdfunding campaigns in our sample, we used zip code to county assignments provided by the US Department of Housing and Urban Development [39]. Each zip code was assigned to a single county that contained the highest proportion of the zip code’s addresses. The total population for each county was obtained from ACS data and used to calculate the total and relative population corresponding to each ADI quartile.

### Statistical Analysis

We used descriptive statistics to summarize the characteristics of our sample, including year of creation, disease categories mentioned, and number of disease categories per campaign.

To estimate the impact of disease categories and ADI quartile on fundraising success, we constructed a generalized linear model (GLM) with the amount of money raised as the dependent variable. The amount raised is the product of the number of donations received and the mean donation amount. We suppose that the number of donations received is Poisson-distributed, and the mean donation amount is gamma-distributed, implying that the amount raised follows a compound Poisson-gamma (ie, Tweedie) distribution. We therefore used a Tweedie GLM with a logarithm link function. The variance power was selected as the value between 1 and 2 that maximized the model likelihood. Disease categories are not mutually exclusive; therefore, each category was coded as present or absent and included in the model. ADI quartile was entered as a categorical variable with the most-deprived quartile serving as the reference group. We also controlled for campaign start year and amount sought, consistent with prior work [17]. The amount sought was observed to be right-skewed and was therefore Box-Cox transformed prior to inclusion. The expected percentage change in the amount raised when a given feature was present was calculated by exponentiating the feature’s β coefficient.

We sought to characterize the donation patterns that underlie differences in fundraising. Because a campaign can increase the amount raised by increasing the number of donations or the mean donation amount, we modeled each of these outcomes separately. To evaluate the impact on the number of donations received, GLMs using Poisson and negative binomial distributions were compared, and the negative binomial model was selected on the basis of a significant likelihood ratio test. The impact on the mean donation amount was evaluated using a GLM with a gamma distribution. Both models used a logarithm link function, and the covariates were identical to the model for the amount raised described above.

Two sensitivity analyses were conducted (Table S2 in Multimedia Appendix 1). First, the amount raised was observed to be highly right-skewed, with a small number of extreme outliers. We hypothesize that these outliers are campaigns that “went viral” and gained widespread attention through social media or traditional media outlets. To evaluate the impact of these extreme values on our modeling strategy, we ran the Tweedie regression using various thresholds above which the amount raised was reassigned to the threshold value (US $1 million and US $100,000-US $500,000 in US $100,000 increments). No important changes in the magnitude or significance of the covariates of interest were observed. Therefore, the uncensored values of the amount sought were used for all analyses.

*P* values <.05 were considered statistically significant. All data analysis was performed using Python (version 3.8.8; Python Software Foundation) and R (version 4.0.3; R Core Team). The source code for the data collection and analysis is available on GitHub [40].

## Results

### Characteristics of Study Sample

Descriptive statistics of our study sample are shown in Table 1. The final sample contained 89,645 unique medical crowdfunding campaigns. Campaigns were created from 2010 to 2020, with only 1.2% (n=1094) of campaigns starting before 2014.

**Table 1.** Characteristics of crowdfunding campaigns in the study sample (n=89,645).

Characteristic	Values, n (%)
Year	
2010‐2013	1094 (1.2)
2014‐2017	46,447 (51.8)
2018‐2020	42,104 (47.0)
Number of unique disease categories per campaign	
0	15,629 (17.4)
1	49,080 (54.7)
2	15,921 (17.8)
3+	9105 (10.1)
Disease category^a^	
Neoplasms	38,221 (43.7)
Injuries and external causes	18,087 (20.7)
Cardiovascular diseases	11,923 (13.6)
Infections	8742 (10.0)
Nervous system diseases	7705 (8.8)
Musculoskeletal diseases	5594 (6.4)
Mental health disorders	4838 (5.5)
Respiratory diseases	4836 (5.5)
Gastrointestinal diseases	4746 (5.4)
Genitourinary diseases	4698 (5.4)
Endocrine diseases	3882 (4.4)

a Disease categories were not mutually exclusive.

Our disease categorization algorithm identified at least one disease category in 82.6% (n=74,016) of campaigns (Table 1). The majority of campaigns (49,080/89,645, 54.7%) mentioned a single disease category, while 27.9% (n=25,026) of campaigns mentioned more than one disease category. In 17.4% (n=15,629) of campaigns, our algorithm did not identify any disease category.

Neoplasms were the most commonly mentioned medical conditions (43.7%, n=38,221 of campaigns), followed by injuries and external causes (20.7% [n=18,087] of campaigns), and cardiovascular diseases (13.6%, n=11,923 of campaigns). Each of the remaining disease categories was detected in 10% or less of campaigns (Table 1). The relative frequency of each disease category was similar each year after 2011 (Figure S2 in Multimedia Appendix 2).

### Overrepresentation of Less-Deprived Areas in Web-Based Crowdfunding

**Figure 1.**
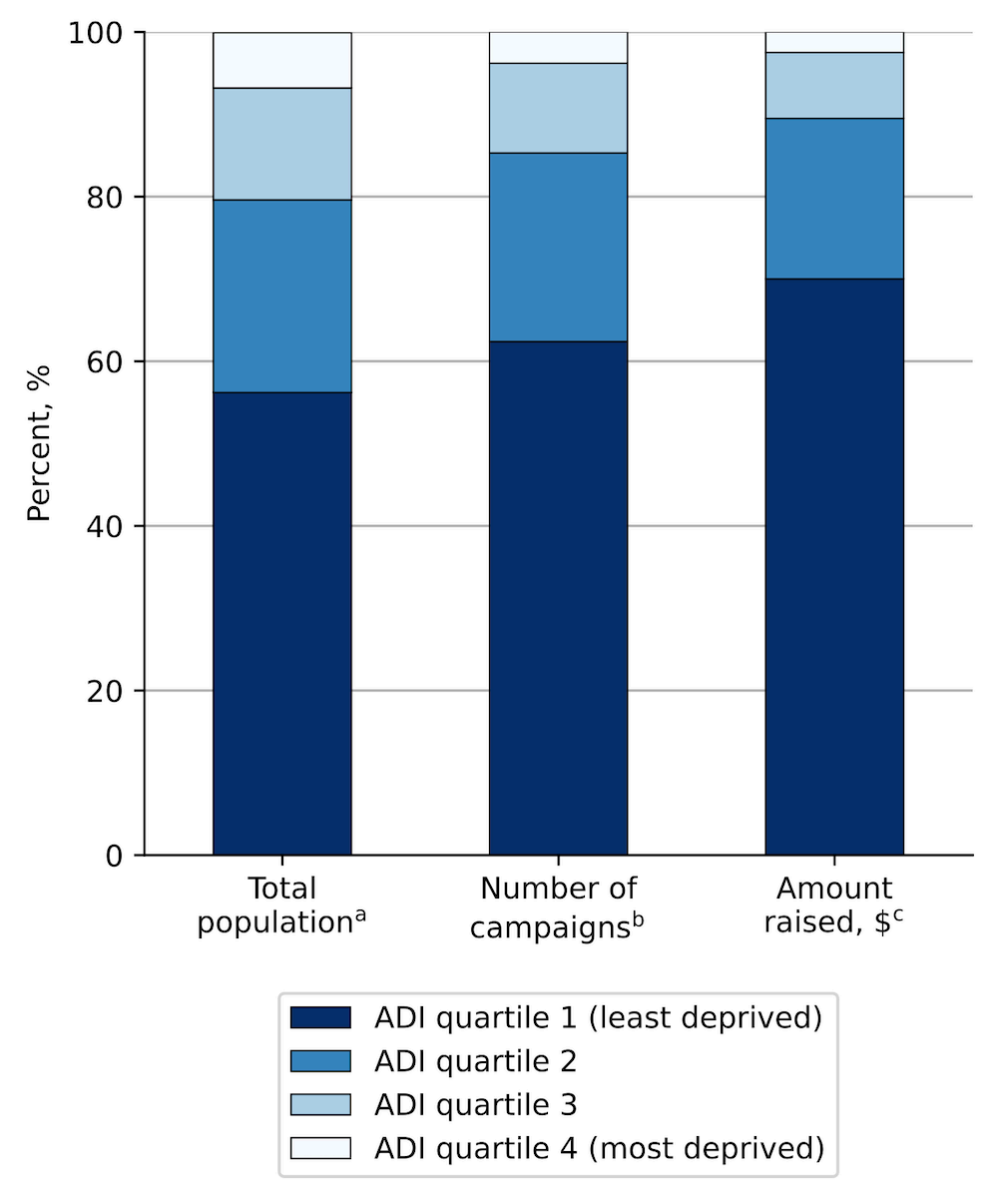
Distribution of campaign frequency and fundraising amount by ADI quartile. ^a^Distribution of United States population according to county-level ADI quartile. ^b^Relative frequency of medical crowdfunding campaigns in our sample based on the ADI quartile of the campaign’s county of origin. ^c^Relative proportion of the total amount raised in our sample according to the ADI quartile of the campaign’s county of origin. ADI: Area Deprivation Index.

The percent of campaigns belonging to each ADI quartile was similar each year after 2011 (Figure S3 in Multimedia Appendix 2). While the least-deprived quartile of counties makes up 56.2% of the population, they account for 62.4% of GFM campaigns (Figure 1). Conversely, the most-deprived quartile of counties makes up 6.7% of the population but only contributed 3.8% of GFM campaigns. In addition, 70% of fundraising in our sample went to campaigns from the least-deprived counties, and only 2.5% went to campaigns from the most-deprived counties.

### Impact of Disease Categories and ADI on Amount Raised

**Figure 2.**
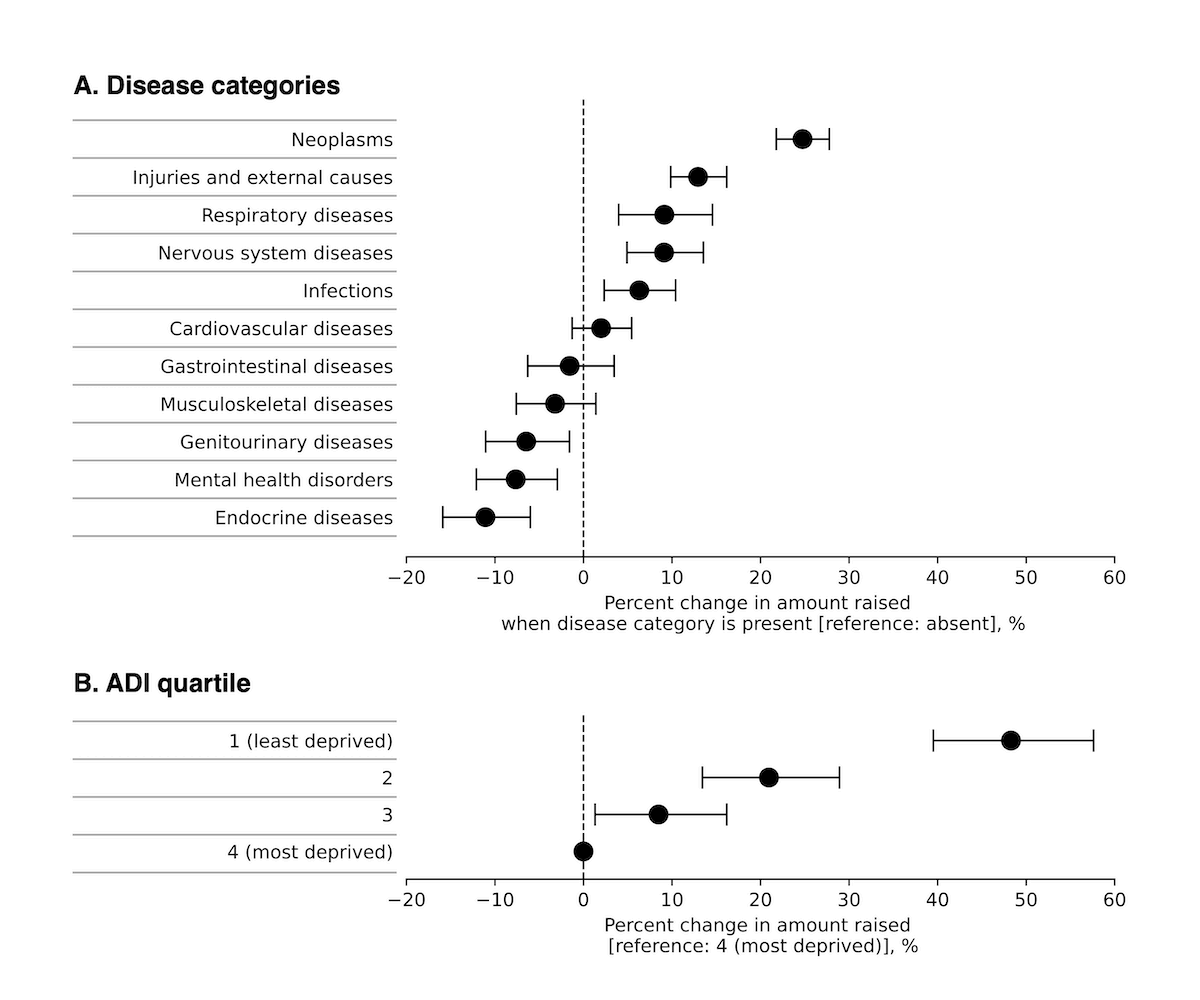
Association of disease categories and ADI with the amount raised per campaign. Tweedie GLM of the amount raised. The model included the displayed variables and was further adjusted for the campaign start year and the amount sought (Box-Cox transformed). The expected percent change in the amount raised was calculated by exponentiating each disease category’s β coefficient. Error bars represent 95% CIs. Disease categories were not mutually exclusive. ADI: Area Deprivation Index.

After adjusting for ADI quartile, campaign start year, and amount sought, we found that most disease categories were statistically significant determinants of the amount of money raised by each campaign (Figure 2, full model outputs are in Table S3 in Multimedia Appendix 2).

Campaigns that mentioned neoplasms raised an average of 24.7% (95% CI 21.8‐27.8) more than those that did not. Campaigns were also more likely to have higher fundraising amounts when they mentioned injuries and external causes (12.9%, 95% CI 9.9‐16.2 increase), respiratory diseases (9.1%, 95% CI 4.0‐14.6 increase), nervous system diseases (9.1%, 95% CI 4.9‐13.5 increase), and infections (6.3%, 95% CI 2.3‐10.4 increase). By contrast, campaigns mentioning endocrine diseases raised 11.1% (95% CI −15.9 to −6.0) less than those that did not. A reduction in the amount raised was also seen for campaigns that mentioned mental health disorders (7.7%, 95% CI −12.1 to −3.0 decrease) and genitourinary diseases (6.5%, 95% CI −11.0 to −1.6 decrease).

The ADI quartile of a campaign’s county of origin was an independent predictor of the amount raised. Adjusting for disease categories mentioned in the campaign, campaign start year, and amount sought, campaigns originating in the least-deprived counties were predicted to earn 48.3% (95% CI 39.5‐57.6) more than counties originating from the most deprived counties (Figure 2). A similar but attenuated relationship between area deprivation and the amount raised was observed for campaigns from intermediate ADI quartiles.

### Impact of Disease Categories and ADI on Number and Size of Donations

We found that the majority of disease categories were significant independent predictors of the number of donations a campaign received, but only 3 out of 11 disease categories (neoplasms, injuries and external causes, and mental health disorders) were significant independent predictors of mean donation amount (Table 2). Similarly, ADI quartile was a strong and statistically significant predictor of the number of donations, but only the most extreme pairwise comparison between the least- and most-deprived counties showed a statistically significant association with the mean donation amount.

**Table 2.** Association of disease categories and Area Deprivation Index (ADI) with donations. The number of donations was modeled using a generalized linear model with a negative binomial distribution. The mean donation amount was modeled using a generalized linear model with a gamma distribution. Both models included the displayed variables and were further adjusted for campaign start year and amount sought (Box-Cox transformed).

Variable	Number of donations		Mean donation amount	
	β (SE)	*P* value	β (SE)	*P* value
Disease category				
Neoplasms	0.135 (0.007)	<.001	0.022 (0.008)	<.001
Injuries and external causes	0.167 (0.008)	<.001	−0.054 (0.009)	<.001
Cardiovascular diseases	0.002 (0.009)	.85	0.009 (0.011)	.42
Infections	0.058 (0.010)	<.001	1.5 × 10^–5^ (9.5 × 10^–3^)	.99
Nervous system diseases	0.046 (0.011)	<.001	0.016 (0.013)	.24
Musculoskeletal diseases	−0.067 (0.013)	<.001	0.023 (0.016)	.15
Mental health disorders	−0.081 (0.014)	<.001	0.052 (0.017)	<.01
Respiratory diseases	0.096 (0.014)	<.001	−0.008 (0.016)	.65
Gastrointestinal diseases	−0.030 (0.014)	.03	0.019 (0.016)	.26
Genitourinary diseases	−0.064 (0.014)	<.001	−0.012 (0.017)	.47
Endocrine diseases	−0.127 (0.015)	<.001	0.013 (0.018)	.47
ADI quartile				
1 (least deprived)	0.333 (0.016)	<.001	0.048 (0.019)	.01
2	0.176 (0.017)	<.001	0.030 (0.020)	.15
3	0.094 (0.018)	<.001	−0.010 (0.022)	.63
4 (most deprived)	Ref^a^	Ref	Ref	Ref

a Reference group.

## Discussion

### Principal Results

In this analysis of web-based medical crowdfunding campaigns, the success of campaigns was influenced by the specific diseases mentioned in the campaign description as well as the socioeconomic deprivation of the county in which the campaign was initially posted. In addition, differences in fundraising were more often explained by the number of donations received rather than the mean donation amount.

Our findings largely corroborate previous reports on the frequency of disease categories in data from GFM. For example, 43.7% of campaigns in our sample raised money for cancer. Among studies that have quantified the frequency of multiple disease categories, cancer was also the most common category, comprising 34.9%‐54.5% of campaigns [8,23]. Similarly, injuries comprised the second most common disease category in our study, consistent with previous work [8,23]. Our sample contained more campaigns about cardiovascular diseases than nervous system diseases, but the opposite was found in other studies [8,23]. These differences may be attributable to how certain medical conditions were categorized (eg, cerebrovascular disease can be reasonably categorized as cardiovascular or neurologic). We also provide estimates for the relative frequency of several disease categories that were previously unexplored in medical crowdfunding, such as mental health disorders, infections, and genitourinary diseases.

After adjusting for other plausible contributors to crowdfunding success, most disease categories described in a crowdfunding campaign were significant predictors of the amount of money raised. In particular, we found that mentioning mental health disorders, endocrine diseases, or genitourinary diseases in the campaign description negatively impacted fundraising success. This trend may reflect perceptions about conditions in these categories. For example, disclosing mental health disorders might invoke stereotypes of the beneficiary being unfit to manage finances [41]. Likewise, endocrine diseases such as diabetes may be viewed as preventable with healthy lifestyle choices and thus undeserving of charitable support [42]. Several studies—mostly small, qualitative, or theoretical—have called attention to the fact that many crowdfunding campaigns present a narrative that emphasizes why the beneficiary is “worthy” or “deserves” financial assistance [22,43-45]. This may be especially challenging for patients with conditions that are commonly associated with personal behaviors, such as diabetes. Here, we present high-quality data that mentioning certain diseases, even when controlling for other important factors, was associated with a lower amount raised, suggesting that potential donors may view these conditions unfavorably.

Our study expands on previous work by characterizing the differences in donation behavior underlying fundraising success. We found that the number of donations seemed to have a greater effect on fundraising success than the average donation amount, both for certain disease categories and area deprivation. Few studies have disaggregated fundraising success into donation number and amount, but our findings largely corroborate those that have. For example, one study using a large sample of medical crowdfunding campaigns from China showed that campaigns about cancer had both a higher number of donations and a higher mean donation amount, but differences in mean donation amount among different disease categories were less pronounced [46]. Another large study using campaigns from GFM found that the number of donations and overall amount raised were lowest in low-income campaigns, and both measures increased in campaigns from higher-income areas, in agreement with our results [35].

Analyzing donation behavior may help explain differences in overall fundraising success across disease categories. For example, we found that mentioning injuries and external causes was associated with a higher amount raised, and a larger number of donations, but a smaller average donation amount. Conversely, mentioning mental health disorders was associated with a lower amount raised, a smaller number of donations, but also a significant increase in the mean donation amount. Although our study was not designed to uncover a precise mechanism for these trends, other work sheds light on potential explanations. One possibility is that the discordance between donation count and donation amount may be unrelated to the content of the campaign and simply reflect the timing of donations. One study of GFM campaigns found that the average donation amount decreases as the number of donors increases [47], and another analysis of medical crowdfunding campaigns from China also found the same trend [48]. In other words, early donations are more generous, and as campaigns accumulate funding, individual contributions become smaller. However, this would not explain our findings that mentioning neoplasms was associated with a higher amount and a higher number of donations, nor would it account for our findings that mentioning endocrine diseases, despite being associated with a smaller number of donations, was not associated with a compensatory increase in donation amount. Therefore, this suggests that there may be some context-specific, or even disease-specific, impact on donation behavior. For example, while there may be bias against donations to campaigns that mention mental health conditions, those who are sympathetic to those campaigns may be especially motivated to donate.

This study highlights the importance of socioeconomic factors in medical crowdfunding participation and success. Relative to their population, we found that less-deprived counties were over-represented in crowdfunding campaigns. Recent work by Kenworthy and Igra [35] analyzed a large sample of GFM campaigns in the United States from 2016 to 2020 and assigned campaigns into quintiles based on the per-capita income of the campaign’s zip code. They found that the highest number of campaigns belonged to middle-income quintiles (rather than the lowest- or highest-income quintiles), which differs slightly from our results but may reflect methodologic differences between analyzing income at the zip code level and a composite ADI at the county level. However, they similarly found that campaigns in the highest income quintile received a proportionally larger share of funds raised, in close agreement with our results. Another study analyzing GFM campaigns from Washington state found that counties with a lower measure of social marginalization tended to have a higher prevalence of campaigns related to COVID-19, but this was not explained by differences in COVID-19 impact (ie, cases), highlighting the unique impact of county-level socioeconomics on participation in medical crowdfunding [49].

Web-based crowdfunding theoretically allows users to reach potential donors beyond their geographic vicinity, but these studies suggest that a campaign’s financial success often reflects the socioeconomic conditions of the campaign’s location. The leading theory for this association is that social networks tend to cluster within socioeconomic groups, rather than span across them [34,50]. Crowdfunding studies in other fields, such as business and entrepreneurship, have similarly found that social capital and networks play an important role in fundraising success [51,52]. To the extent that many campaigns rely on social networks as their primary donor pool, the socioeconomic composition of one’s social network will likely remain an important part of fundraising outcomes.

Given the potential barriers to accessing web-based crowdfunding in more deprived counties, our findings should not be interpreted as representative of the demand for medical crowdfunding or out-of-pocket cost burden. Nevertheless, our results demonstrate that the socioeconomic backdrop of a campaign’s location has a significant impact on fundraising success, even when adjusting for the specific diseases that are described in the campaign. Similar to the effect of medical conditions on the amount raised, this relationship was more consistently explained by a higher number of donations, rather than a larger value of the average donation. Taken together, these results show that web-based crowdfunding for medical expenses channels funds toward specific diseases and beneficiaries in less-deprived areas. This suggests that relying on charitable giving may selectively reward certain diseases, thereby failing to systematically address gaps in coverage or financial support.

### Limitations

This study has several limitations. First, we used data from a single crowdfunding platform, which may limit the generalizability of our findings. Second, because campaigns can be voluntarily removed from GFM at any time, we were only able to sample campaigns that were available on the GFM sitemap at the time of data collection, which could introduce selection bias (MultimediaAppendices 1  2 contain further discussion). Third, our disease identification algorithm could not distinguish medical conditions that were only incidentally mentioned in the campaign description from those that were directly relevant to the campaign, nor could it capture the severity of different conditions. Fourth, our algorithm had lower precision (positive predictive value) and recall (sensitivity) for detecting the presence of certain disease categories, such as gastrointestinal diseases and nervous system diseases [38]. This could lead to reporting a falsely low frequency of these disease categories. Fifth, we report the amount raised as the primary measure of campaign success rather than the percentage of the goal raised, even though the latter might be more relevant to individual campaign organizers. However, we adjusted for the amount sought in our multivariable analysis to account for the impact of the goal amount on fundraising behavior. Sixth, many factors are likely to contribute to a campaign’s fundraising success that we did not measure, including the semantic features of the campaign description and the social capital of the campaign organizer or beneficiary [17,50,53]. Seventh, non-English campaigns were excluded for technical purposes to ensure that the natural language processing component of the disease identification algorithm would function as intended, which may impact the generalizability of our findings to communities that prefer to communicate in other languages. Last, we excluded several categories of medical conditions from our analysis due to inadequate data while developing our disease identification algorithm; these include obstetric and gynecologic conditions, diseases of the sense organs, hematologic and immune disorders, and chromosomal abnormalities.

### Conclusions

The medical conditions mentioned in crowdfunding campaigns matter for the fundraising success of the campaign. Campaigns raising money for neoplasms were the most frequent and financially successful, and some conditions adversely impacted fundraising. Regardless of the specific diseases mentioned in the campaign, socioeconomic factors had a significant impact on fundraising success.

## Supplementary material

10.2196/72475 Multimedia Appendix 1 Additional online material for methods.

10.2196/72475 Multimedia Appendix 2 Additional online material for results.

10.2196/72475 Checklist 1 STROBE Statement—Checklist of items that should be included in reports of cross-sectional studies.

## Abbreviations

ACS: American Community Survey
ADI: Area Deprivation Index
GFM: GoFundMe
GLM: generalized linear model
*ICD-10-CM*: *International Classification of Diseases, Tenth Revision, Clinical Modification*
STROBE: Strengthening the Reporting of Observational Studies in Epidemiology

## References

[1] Norton M, Pollitz K, Claxton G (2016). The burden of medical debt: results from the kaiser family foundation/new york times medical bills survey. Kaiser Family Foundation.

[2] Cha AE, Cohen RA (2020). Problems paying medical bills, 2018. NCHS Data Brief.

[3] Rosenthal MB (2021). The growing problem of out-of-pocket costs and affordability in employer-sponsored insurance. JAMA.

[4] Himmelstein DU, Lawless RM, Thorne D, Foohey P, Woolhandler S (2019). Medical bankruptcy: still common despite the affordable care act. Am J Public Health.

[5] Successful fundraisers start here. GoFundMe.

[6] McClanahan C People are raising $650 million on gofundme each year to attack rising healthcare costs. Forbes.

[7] Bluth R (2019). GoFundMe CEO: ‘Gigantic Gaps’ In health system showing up in crowdfunding. Kaiser Health News.

[8] Angraal S, Zachariah AG, Raaisa R (2021). Evaluation of internet-based crowdsourced fundraising to cover health care costs in the United States. JAMA Netw Open.

[9] Thomas HS, Lee AW, Nabavizadeh B (2021). Characterizing online crowdfunding campaigns for patients with kidney cancer. Cancer Med.

[10] Panjwani A, Xiong H (2023). The causes and consequences of medical crowdfunding. J Econ Behav Organ.

[11] Snyder J, Turner L, Crooks VA (2018). Crowdfunding for unproven stem cell-based interventions. JAMA.

[12] Vox F, Folkers KM, Turi A, Caplan AL (2018). Medical crowdfunding for scientifically unsupported or potentially dangerous treatments. JAMA.

[13] Snyder J, Cohen IG (2019). Medical crowdfunding for unproven medical treatments: should Gofundme become a gatekeeper?. Hastings Cent Rep.

[14] Snyder J, Zenone M, Crooks V, Schuurman N (2020). What medical crowdfunding campaigns can tell us about local health system gaps and deficiencies: exploratory analysis of British Columbia, Canada. J Med Internet Res.

[15] Cohen AJ, Brody H, Patino G (2019). Use of an online crowdfunding platform for unmet financial obligations in cancer care. JAMA Intern Med.

[16] Dieleman JL, Cao J, Chapin A (2020). US health care spending by payer and health condition, 1996-2016. JAMA.

[17] Silver ER, Truong HQ, Ostvar S, Hur C, Tatonetti NP (2020). Association of neighborhood deprivation index with success in cancer care crowdfunding. JAMA Netw Open.

[18] Loeb S, Taneja S, Walter D, Zweifach S, Byrne N (2018). Crowdfunding for prostate cancer and breast cancer. BJU Int.

[19] Snyder J, Caulfield T (2019). Patients’ crowdfunding campaigns for alternative cancer treatments. Lancet Oncol.

[20] Rajwa P, Hopen P, Wojnarowicz J (2022). Online crowdfunding for urologic cancer care. Cancers (Basel).

[21] O’Connor RM, Huang DS, Rimel BJ (2024). Unmet financial needs among patients crowdfunding to support gynecologic cancer care. Gynecol Oncol.

[22] Zhang X, Tao X, Ji B, Wang R, Sörensen S (2023). The success of cancer crowdfunding campaigns: project and text analysis. J Med Internet Res.

[23] Saleh SN, Ajufo E, Lehmann CU, Medford RJ (2020). A comparison of online medical crowdfunding in Canada, the UK, and the US. JAMA Netw Open.

[24] Snyder J, Turner L (2019). Crowdfunding for stem cell-based interventions to treat neurologic diseases and injuries. Neurology (ECronicon).

[25] Galvin C, Yang W, Saadi A (2023). Evaluation of crowdsourced fundraising to cover health care costs for neurological conditions in the US. JAMA Neurol.

[26] Defilippis EM, Mehta A, Alkhunaizi FA (2024). The wallet biopsy: medical crowdfunding for heart transplantation. J Card Fail.

[27] Joseph Mattingly T, Li K, Ng A, Ton-Nu TL, Owens J (2021). Exploring patient-reported costs related to hepatitis c on the medical crowdfunding page GoFundMe®. Pharmacoecon Open.

[28] Rajwa P, Hopen P, Mu L (2020). Online crowdfunding response to coronavirus disease 2019. J Gen Intern Med.

[29] Kassamali B, Okoli T, Lim S (2024). Crowdfunding to compensate for financial burdens of patients with systemic sclerosis. Arch Dermatol Res.

[30] Sloan CE, Campagna A, Tu K, Doerstling S, Davis JK, Ubel PA (2023). Online crowdfunding campaigns for diabetes-related expenses. Ann Intern Med.

[31] Larkin J, Foley L, Smith SM, Harrington P, Clyne B (2021). The experience of financial burden for people with multimorbidity: a systematic review of qualitative research. Health Expect.

[32] Carman KG, Liu J, White C (2020). Accounting for the burden and redistribution of health care costs: who uses care and who pays for it. Health Serv Res.

[33] van Duynhoven A, Lee A, Michel R (2019). Spatially exploring the intersection of socioeconomic status and Canadian cancer-related medical crowdfunding campaigns. BMJ Open.

[34] Yang S, Ke X, Cheng C, Bian Y (2023). A matter of life and death: the power of personal networks for medical crowdfunding performance. Soc Sci Med.

[35] Kenworthy N, Igra M (2022). Medical crowdfunding and disparities in health care access in the United States, 2016‒2020. Am J Public Health.

[36] von Elm E, Altman DG, Egger M (2007). Strengthening the reporting of observational studies in epidemiology (STROBE) statement: guidelines for reporting observational studies. BMJ.

[37] Joulin A, Grave E, Bojanowski P, Douze M, Jégou H, Mikolov T (2016). FastText.zip: compressing text classification models. arXiv.

[38] Doerstling SS, Akrobetu D, Engelhard MM, Chen F, Ubel PA (2022). A disease identification algorithm for medical crowdfunding campaigns: validation study. J Med Internet Res.

[39] HUD USPS ZIP code crosswalk files. HUD User.

[40] Doerstling S Sdoerstling/medical_crowdfunding_analysis. GitHub.

[41] Harper A, Clayton A, Bailey M, Foss-Kelly L, Sernyak MJ, Rowe M (2015). Financial health and mental health among clients of a community mental health center: making the connections. PS (Wash DC).

[42] Vishwanath A (2014). Negative public perceptions of juvenile diabetics: applying attribution theory to understand the public’s stigmatizing views. Health Commun.

[43] Berliner LS, Kenworthy NJ (2017). Producing a worthy illness: personal crowdfunding amidst financial crisis. Soc Sci Med.

[44] Lukk M, Schneiderhan E, Soares J (2018). Worthy? crowdfunding the Canadian health care and education sectors. Can Rev Sociol.

[45] Paulus TM, Roberts KR (2018). Crowdfunding a “Real-life Superhero”: the construction of worthy bodies in medical campaign narratives. Discourse Context Media.

[46] Zhang S, Zhang Q, Wang M, Tang X, Lu X, Huang W (2025). Key drivers of medical crowdfunding success: a comprehensive analysis of 84,712 projects. Humanit Soc Sci Commun.

[47] Ren J, Raghupathi V, Raghupathi W (2020). Understanding the dimensions of medical crowdfunding: a visual analytics approach. J Med Internet Res.

[48] Wang M, Cai M, Guo S (2024). Large-scale medical crowdfunding data reveal determinants and preferences of donation behaviors. IEEE Trans Comput Soc Syst.

[49] Luchsinger C, Kenworthy N, Igra M, Jung JK (2024). Inequity in Washington state covid-19-related crowdfunding. Soc Sci Humanit Open.

[50] Snyder J (2016). Crowdfunding for medical care: ethical issues in an emerging health care funding practice. Hastings Cent Rep.

[51] Santos SC, Costa S, Shafi K, Neumeyer X (2024). Tweeting to triumph: gender differences in harnessing social media for crowdfunding. J Small Bus Manag.

[52] Belleflamme P, Lambert T, Schwienbacher A (2014). Crowdfunding: tapping the right crowd. J Bus Ventur.

[53] Barcelos CA, Budge SL (2019). Inequalities in crowdfunding for transgender health care. Transgend Health.

