# Supplementary material for: Impact of Medical Conditions and Area Deprivation on Fundraising Success in Online Crowdfunding: Cross-Sectional Study
Source: J Med Internet Res. 2025 Jul 29;27:e72475. doi: 10.2196/72475 (PMC12306843; doi:10.2196/72475)
Supplement: Multimedia Appendix 1 [file jmir-v27-e72475-s001.pdf]

Multimedia Appendix 1

Doerstling SS, et al. “Impact of Medical Conditions and Area Deprivation on Fundraising Success in Online Crowdfunding”

Table of Contents

Supplemental text S1. Discussion of selection bias in the study sample……………………………………………p2
Supplemental text S2. Discussion of GFM policy limiting receipt of donations……………………………………p3
Figure S1. Association between campaign age and percent of 12-month earnings raised……………………....p4
Supplemental text S3. Calculation of area deprivation index…………………………………………………………...p5
Table S1. PCA of census variables…………………………………………………………………………………………...p6
Table S2. Sensitivity analysis of amount raised thresholds and amount sough transformation in Tweedie GLMs……...p7
References………...p8

Supplemental text S1. Discussion of selection bias in the study sample.
If we define survival for a given crowdfunding campaign as the condition of being created before our data collection and remaining on the GoFundMe (GFM) sitemap at the time of our data collection, then our random sample could be subject to survivorship bias. Specifically, campaigns that were created before our data collection but were then removed from the GFM sitemap for any reason would fail to survive and therefore be excluded from the population that was randomly sampled. This would introduce selection bias if there were systematic differences between the campaigns that survived and those that failed to survive. Although we do not have prospective data on the survival of crowdfunding campaigns, we offer the following discussion as context for considering the possible impact of selection bias in our sample.

First, it is important to note that by default, campaigns "remain live until [campaign organizers] choose to turn off donations or remove the campaign altogether." (www.gofundme.com, "Answers to Common Fundraising Questions", December 2021). Furthermore, both GFM and campaign organizers have a financial incentive to keep campaigns active because both entities raise money from donations. GFM notes that "most organizers leave their campaigns active indefinitely to refer back to the kind comments and support they received." (www.gofundme.com, "Answers to Common Fundraising Questions", December 2021). Campaigns that have met their fundraising goal remain active, and the financial incentives for GFM and campaign organizers persist after the fundraising goal has been met. GFM states that "[campaigns] will be able to accept donations even after your goal is reached." (www.gofundme.com, "Answers to Common Fundraising Questions", December 2021). This is supported by the fact that at the time of our data collection, 14.2% of campaigns in our sample had raised more than 100% of the amount sought.

There are several possible reasons why a campaign may have been made unavailable to our data collection. Broadly, campaigns could have been intentionally inactivated by either GFM or campaign organizers. GFM might remove campaigns that violate their terms of use. Campaign organizers might remove campaigns to limit private information on the internet or once a beneficiary has passed away (though we have observed many posthumous medical crowdfunding campaigns in the course of this project). However, as previously noted, neither entity has a financial incentive to remove campaigns.

In summary, while we do not have strong reason to believe that campaigns that failed to survive until our data collection were systematically different from those that survived, we cannot exclude the contribution of survivorship bias in the study sample.

Supplemental text S2. Discussion of GFM policy limiting receipt of donations.
GFM policy states that "fundraisers that have not received donations or posted updates in 365 days will be made inactive." (www.gofundme.com, Reactivating your fundraiser, December 2021). Once a campaign is inactive, it cannot receive donations unless reactivated by the campaign organizer(s). We do not have an exact record of when this policy was implemented. Theoretically, if this policy came into effect during the years included in our data collection, then campaigns created before the policy was implemented may have been able to raise additional funds, even if they met criteria for campaign inactivity, compared with campaigns created within one year of policy implementation.

To characterize the relationship between campaign age and fundraising activity, we selected campaigns that were created at least 12 months before our data collection (n=66,927 campaigns) and calculated each campaign's total earnings at 12 months. We found that an average of 80% of 12-month earnings was raised in the first month alone, and campaigns raised an average of 95% of 12-month earnings by 5 months (eFigure 1 below). These findings suggest that if campaigns in our sample were differentially exposed to the policy limiting donations after 365 days of inactivity, that the impact on the amount raised would likely be marginal and not significantly bias higher earnings toward campaigns created before policy implementation.

Figure S1. Association between campaign age and percent of 12-month earnings raised.
(n=66,927 campaigns that were created at least 12 months before August 2020)

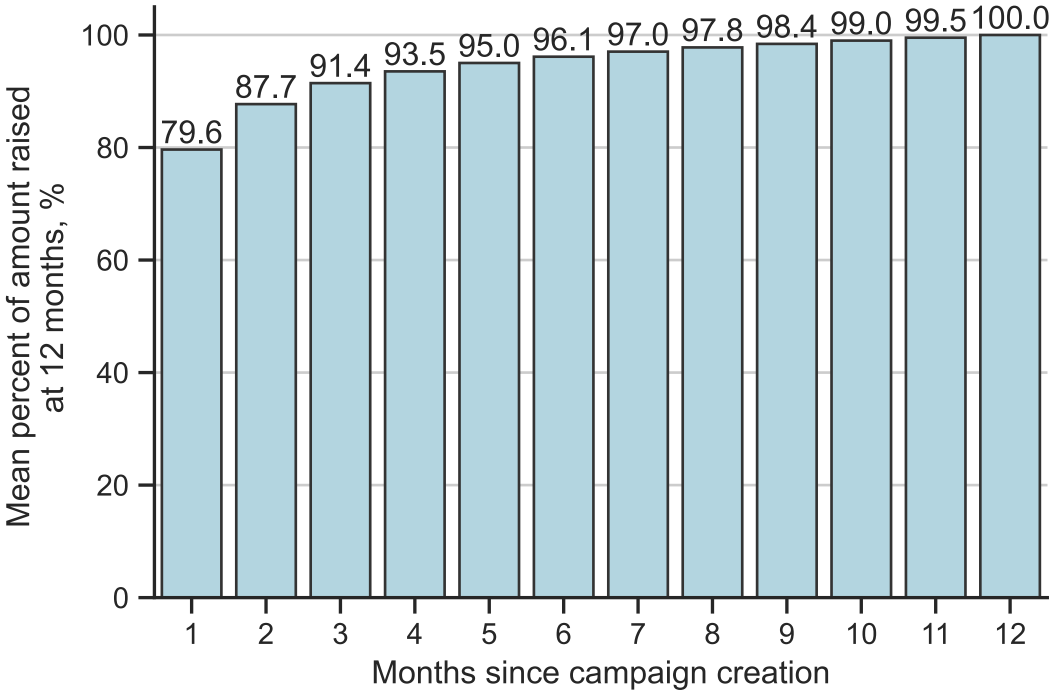

Supplemental text S3. Calculation of area deprivation index.
An area deprivation index was calculated using the same methods described by Silver and colleagues[1]. The only difference was using data from 2019, rather than 2017, for the American Community Survey. This resulted in the same set of variables reaching the absolute value threshold of 0.25 in the initial principal component analysis (PCA). The percent of variance explained by each round of PCA was slightly less than Silver and colleagues. Complete factor loadings by census variable and total variance explained are shown in Table S1 below.

Table S1. PCA of census variables.

Variable Name	Factor loading: PCA round 1	Factor loading: PCA round 2
Unemployment rate	0.325	0.313
Poverty rate	0.406	0.431
Percent without health insurance	0.253	0.271
Home ownership rate	-0.094	NA
Percent with greater than 1 person per room	0.187	NA
Percent without a car	0.233	NA
Percent vacant units	0.158	NA
Percent with high school education	-0.2636	-0.352
Percent with internet access	-0.364	-0.418
Percent Black	0.200	NA
Percent single parent	0.145	NA
Percent with annual income less than $35,000	0.404	0.452
Percent on public assistance	0.176	NA
Percent in management, arts, and science occupations	-0.293	-0.373
Variance Explained	32.9%	55.2%

Table S2. Sensitivity analysis of amount raised thresholds and amount sough transformation in Tweedie GLMs.

Distribution	Tweedie (index parm = 1.72)			Tweedie (index parm = 1.72)			Tweedie (index parm = 1.72)			Tweedie (index parm = 1.72)			Tweedie (index parm = 1.72)			Tweedie (index parm = 1.72)			Tweedie (index parm = 1.72)		
**Dependent variable**	**Amount raised**			**Amount raised capped at $1,000,000**			**Amount raised capped at $500,000**			**Amount raised capped at $400,000**			**Amount raised capped at $300,000**			**Amount raised capped at $200,000**			**Amount raised capped at $100,000**		
**Amount sought**	**Raw**			**Raw**			**Raw**			**Raw**			**Raw**			**Raw**			**Raw**		
**Disease categories (present, reference: absent)**	**Beta**	**Z**	**p value**	**Beta**	**Z**	**p value**	**Beta**	**Z**	**p value**	**Beta**	**Z**	**p value**	**Beta**	**Z**	**p value**	**Beta**	**Z**	**p value**	**Beta**	**Z**	**p value**
Cardiovascular diseases	0.0764	3.236	0.001	0.0596	2.806	0.005	0.0519	2.714	0.007	0.0494	2.657	0.008	0.0477	2.662	0.008	0.0488	2.892	0.004	0.0574	3.816	<0.001
Endocrine diseases	-0.1002	-2.506	0.012	-0.0925	-2.579	0.01	-0.1054	-3.261	0.001	-0.1073	-3.414	0.001	-0.1113	-3.672	<0.001	-0.1156	-4.043	<0.001	-0.1134	-4.45	<0.001
Gastrointestinal diseases	-0.0015	-0.041	0.967	0.0014	0.044	0.965	-0.0024	-0.084	0.933	-0.0036	-0.13	0.897	-0.0039	-0.145	0.885	-0.0062	-0.243	0.808	-0.0087	-0.382	0.703
Genitourinary diseases	-0.0433	-1.193	0.233	-0.0392	-1.205	0.228	-0.0332	-1.133	0.257	-0.0349	-1.226	0.22	-0.0385	-1.4	0.161	-0.0387	-1.494	0.135	-0.0335	-1.451	0.147
Infections	0.1245	4.614	<0.001	0.1310	5.411	<0.001	0.1353	6.216	<0.001	0.1336	6.316	<0.001	0.1319	6.462	<0.001	0.1305	6.781	<0.001	0.1290	7.522	<0.001
Injuries and external causes	0.2179	10.842	<0.001	0.2220	12.308	<0.001	0.2275	14.022	<0.001	0.2287	14.513	<0.001	0.2287	15.048	<0.001	0.2276	15.889	<0.001	0.2191	17.146	<0.001
Mental health disorders	-0.0690	-1.936	0.053	-0.0663	-2.075	0.038	-0.0614	-2.135	0.033	-0.0595	-2.132	0.033	-0.0589	-2.187	0.029	-0.0699	-2.75	0.006	-0.0782	-3.448	0.001
Musculoskeletal diseases	-0.0562	-1.668	0.095	-0.0536	-1.773	0.076	-0.0489	-1.799	0.072	-0.0469	-1.778	0.075	-0.0490	-1.924	0.054	-0.0490	-2.041	0.041	-0.0452	-2.111	0.035
Neoplasms	0.3301	19.65	<0.001	0.3331	22.098	<0.001	0.3401	25.073	<0.001	0.3423	25.975	<0.001	0.3440	27.072	<0.001	0.3471	28.987	<0.001	0.3546	33.23	<0.001
Nervous system diseases	0.1397	4.912	<0.001	0.1437	5.632	<0.001	0.1490	6.494	<0.001	0.1510	6.775	<0.001	0.1524	7.091	<0.001	0.1539	7.598	<0.001	0.1547	8.568	<0.001
Respiratory diseases	0.1955	5.634	<0.001	0.1495	4.774	<0.001	0.1252	4.429	<0.001	0.1212	4.413	<0.001	0.1181	4.456	<0.001	0.1091	4.363	<0.001	0.1003	4.497	<0.001
**ADI quartile**																					
1 (least deprived)	0.5379	12.258	<0.001	0.5346	13.578	<0.001	0.5297	14.96	<0.001	0.5279	15.351	<0.001	0.5292	15.951	<0.001	0.5304	16.962	<0.001	0.5200	18.672	<0.001
2	0.2640	5.745	<0.001	0.2648	6.421	<0.001	0.2616	7.054	<0.001	0.2603	7.227	<0.001	0.2630	7.568	<0.001	0.2676	8.173	<0.001	0.2660	9.121	<0.001
3	0.1058	2.14	0.032	0.1067	2.403	0.016	0.1066	2.672	0.008	0.1066	2.75	0.006	0.1105	2.955	0.003	0.1175	3.336	0.001	0.1186	3.78	<0.001
4 (most deprived, reference)	REF	REF	REF	REF	REF	REF	REF	REF	REF	REF	REF	REF	REF	REF	REF	REF	REF	REF	REF	REF	REF
**Year**	-0.0747	-18.992	<0.001	-0.0739	-20.924	<0.001	-0.0738	-23.219	<0.001	-0.0738	-23.918	<0.001	-0.0743	-24.963	<0.001	-0.0756	-26.963	<0.001	-0.0785	-31.404	<0.001
**Amount sought**	0.0000	0.972	0.331	0.0000	0.621	0.535	0.0000	0.325	0.745	0.0000	0.229	0.819	0.0000	0.1	0.92	0.0000	-0.114	0.91	0.0000	-0.479	0.632
**Intercept**	158.8015	20.009	<0.001	157.1020	22.056	<0.001	156.8679	24.477	<0.001	156.9795	25.213	<0.001	157.9555	26.305	<0.001	160.5909	28.386	<0.001	166.3857	32.999	<0.001

Distribution	Tweedie (index parm = 1.72)			Tweedie (index parm = 1.72)			Tweedie (index parm = 1.72)			Tweedie (index parm = 1.72)			Tweedie (index parm = 1.72)			Tweedie (index parm = 1.72)			Tweedie (index parm = 1.72)		
**Dependent variable**	**Amount raised**			**Amount raised capped at $1,000,000**			**Amount raised capped at $500,000**			**Amount raised capped at $400,000**			**Amount raised capped at $300,000**			**Amount raised capped at $200,000**			**Amount raised capped at $100,000**		
**Amount sought**	**Box-cox transformed**			**Box-cox transformed**			**Box-cox transformed**			**Box-cox transformed**			**Box-cox transformed**			**Box-cox transformed**			**Box-cox transformed**		
**Disease categories (present, reference: absent)**	**Beta**	**Z**	**p value**	**Beta**	**Z**	**p value**	**Beta**	**Z**	**p value**	**Beta**	**Z**	**p value**	**Beta**	**Z**	**p value**	**Beta**	**Z**	**p value**	**Beta**	**Z**	**p value**
Cardiovascular diseases	0.0197	1.17	0.242	0.0167	0.997	0.319	0.0151	0.914	0.361	0.0146	0.883	0.377	0.0143	0.873	0.383	0.0152	0.936	0.349	0.0205	1.315	0.189
Endocrine diseases	-0.1174	-4.13	<0.001	-0.1161	-4.11	<0.001	-0.1189	-4.25	<0.001	-0.1192	-4.273	<0.001	-0.1198	-4.317	<0.001	-0.1201	-4.376	<0.001	-0.1163	-4.415	<0.001
Gastrointestinal diseases	-0.0157	-0.619	0.536	-0.0151	-0.599	0.549	-0.0159	-0.637	0.524	-0.0161	-0.647	0.518	-0.016	-0.649	0.516	-0.0169	-0.694	0.488	-0.0178	-0.761	0.447
Genitourinary diseases	-0.0669	-2.599	0.009	-0.0659	-2.579	0.01	-0.0643	-2.541	0.011	-0.0649	-2.571	0.01	-0.0661	-2.632	0.008	-0.066	-2.657	0.008	-0.0627	-2.631	0.009
Infections	0.0611	3.182	0.001	0.0626	3.279	0.001	0.0636	3.368	0.001	0.0631	3.347	0.001	0.0627	3.342	0.001	0.0629	3.395	0.001	0.0641	3.605	<0.001
Injuries and external causes	0.1217	8.487	<0.001	0.1228	8.62	<0.001	0.1244	8.822	<0.001	0.1248	8.878	<0.001	0.1249	8.925	<0.001	0.1246	9.005	<0.001	0.1221	9.195	<0.001
Mental health disorders	-0.0796	-3.136	0.002	-0.079	-3.134	0.002	-0.0778	-3.119	0.002	-0.0774	-3.111	0.002	-0.0771	-3.113	0.002	-0.08	-3.269	0.001	-0.0812	-3.455	0.001
Musculoskeletal diseases	-0.0326	-1.367	0.172	-0.0321	-1.353	0.176	-0.031	-1.321	0.186	-0.0305	-1.305	0.192	-0.0312	-1.341	0.18	-0.031	-1.347	0.178	-0.032	-1.447	0.148
Neoplasms	0.2211	18.346	<0.001	0.2221	18.548	<0.001	0.2243	18.919	<0.001	0.225	19.043	<0.001	0.2258	19.204	<0.001	0.2276	19.574	<0.001	0.2332	20.9	<0.001
Nervous system diseases	0.0871	4.314	<0.001	0.088	4.389	<0.001	0.0895	4.508	<0.001	0.0901	4.553	<0.001	0.0905	4.595	<0.001	0.091	4.675	<0.001	0.0923	4.938	<0.001
Respiratory diseases	0.0874	3.519	<0.001	0.0788	3.19	0.001	0.074	3.024	0.002	0.0734	3.008	0.003	0.073	3.007	0.003	0.0709	2.953	0.003	0.0698	3.028	0.002
**ADI quartile**																					
1 (least deprived)	0.394	12.684	<0.001	0.3937	12.757	<0.001	0.3932	12.867	<0.001	0.393	12.902	<0.001	0.3935	12.981	<0.001	0.3939	13.143	<0.001	0.3903	13.576	<0.001
2	0.1902	5.848	<0.001	0.1905	5.896	<0.001	0.1902	5.944	<0.001	0.19	5.959	<0.001	0.1908	6.014	<0.001	0.1924	6.132	<0.001	0.1921	6.382	<0.001
3	0.0814	2.327	0.02	0.0817	2.349	0.019	0.0819	2.378	0.017	0.0819	2.388	0.017	0.0829	2.429	0.015	0.0848	2.512	0.012	0.0846	2.613	0.009
4 (most deprived, reference)	REF	REF	REF	REF	REF	REF	REF	REF	REF	REF	REF	REF	REF	REF	REF	REF	REF	REF	REF	REF	REF
**Year**	-0.0718	-25.531	<0.001	-0.0717	-25.638	<0.001	-0.0717	-25.896	<0.001	-0.0717	-25.989	<0.001	-0.0719	-26.183	<0.001	-0.0724	-26.683	<0.001	-0.0737	-28.304	<0.001
**Amount sought**	0.1999	87.307	<0.001	0.1992	87.571	<0.001	0.1983	87.954	<0.001	0.1979	88.058	<0.001	0.1973	88.208	<0.001	0.196	88.55	<0.001	0.1918	90.126	<0.001
**Intercept**	150.4368	26.494	<0.001	150.1141	26.608	<0.001	150.1608	26.878	<0.001	150.2245	26.975	<0.001	150.5916	27.174	<0.001	151.6957	27.689	<0.001	154.3642	29.36	<0.001

References

1. Silver ER, Truong HQ, Ostvar S, Hur C, Tatonetti NP. Association of Neighborhood Deprivation Index With Success in Cancer Care Crowdfunding. *JAMA Netw Open*. 2020;3(12):e2026946. doi:10.1001/jamanetworkopen.2020.26946
