# Supplementary material for: Impact of Medical Conditions and Area Deprivation on Fundraising Success in Online Crowdfunding: Cross-Sectional Study
Source: J Med Internet Res. 2025 Jul 29;27:e72475. doi: 10.2196/72475 (PMC12306843; doi:10.2196/72475)
Supplement: Multimedia Appendix 2 [file jmir-v27-e72475-s002.pdf]

Multimedia Appendix 2

Doerstling SS, et al. "Impact of Medical Conditions and Area Deprivation on Fundraising Success in Online Crowdfunding"

<u>Table of Contents</u>

Figure S2. Relative frequency of disease category by year………………………………………………………………p2
Figure S3. Relative frequency of ADI quartile by year…………………………………………..……………………………p3
Table S3. Generalized linear model of amount raised……………………………………………………………………...p4

Figure S2. Relative frequency of disease category by year.

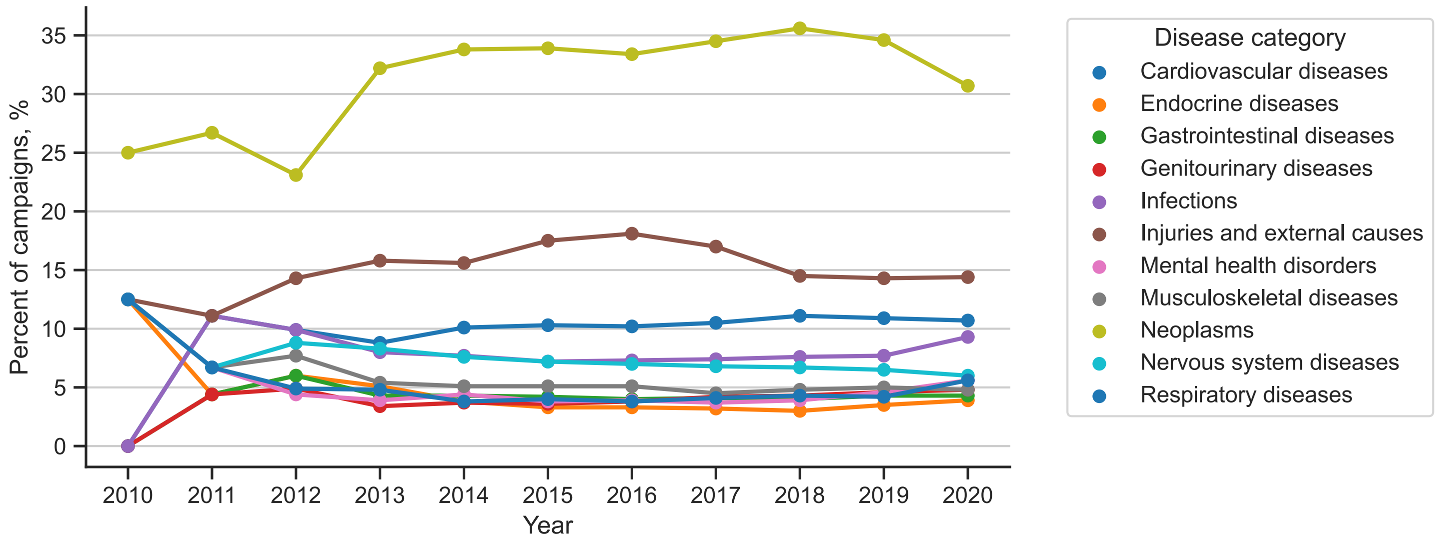

Figure S3. Relative frequency of ADI quartile by year.

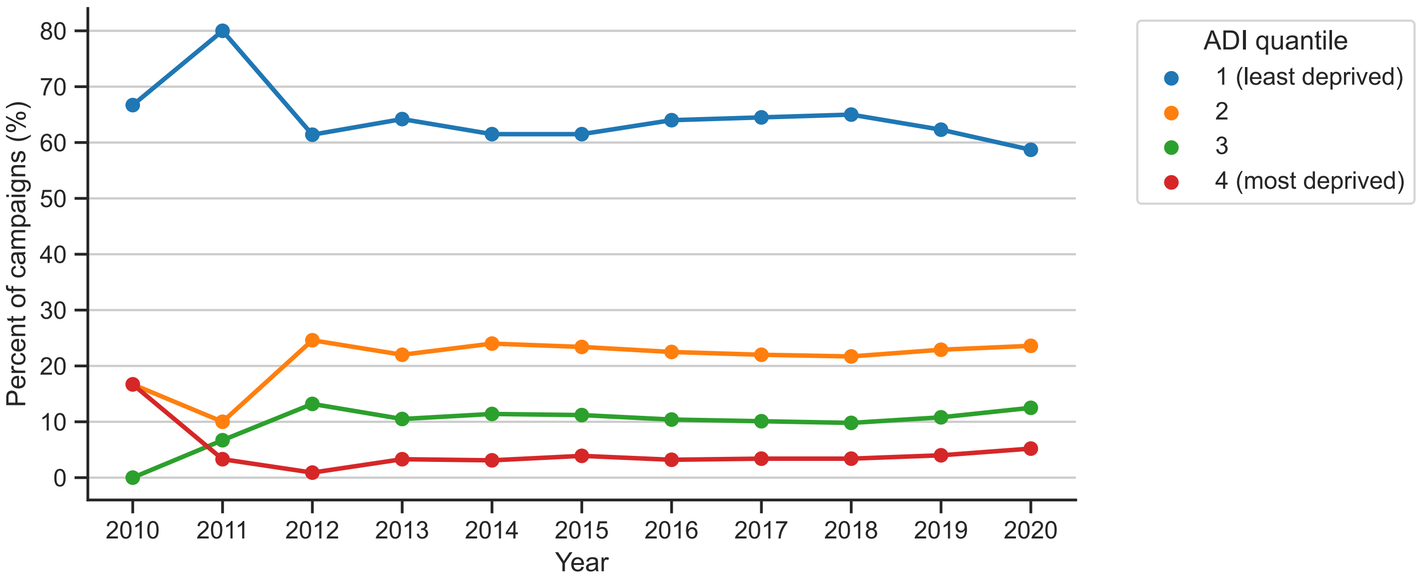

Table S3. Generalized linear model of amount raised.

Variable		Beta (SE[a])	Expected percent change in amount raised, mean (95% CI [b])	*P* value
**Disease category (present, reference: absent)**				
	Cardiovascular diseases	0.02 (0.02)	2.0 (-1.3, 5.4)	.242
	Endocrine diseases	-0.12 (0.03)	-11.1 (-15.9, -6.0)	<.001
	Gastrointestinal diseases	-0.02 (0.02)	-1.6 (-6.3, 3.5)	.536
	Genitourinary diseases	-0.07 (0.03)	-6.5 (-11.0, -1.6)	.009
	Infections	0.06 (0.02)	6.3 (2.3, 10.4)	.001
	Injuries and external causes	0.12 (0.01)	12.9 (9.9, 16.2)	<.001
	Mental health disorders	-0.08 (0.02)	-7.7 (-12.1, -3.0)	.002
	Musculoskeletal diseases	-0.03 (0.02)	-3.2 (-7.6, 1.4)	.172
	Neoplasms	0.22 (0.01)	24.7 (21.8, 27.8)	<.001
	Nervous system diseases	0.09 (0.02)	9.1 (4.9, 13.5)	<.001
	Respiratory diseases	0.09 (0.02)	9.1 (4.0, 14.6)	<.001
**ADI[c] quartile**				
	1 (least deprived)	0.39 (0.03)	48.3 (39.5, 57.6)	<.001
	2	0.19 (0.03)	20.9 (13.4, 28.9)	<.001
	3	0.08 (0.04)	8.5 (1.3, 16.2)	.020
	4 (most deprived)	Reference	Reference	Reference
**Year**		-0.07 (0.0)	-6.9 (-7.4, -6.4)	<.001
**Amount sought[d]**		0.2 (0.0)	22.1 (21.5, 22.6)	<.001
**Intercept**		150.44 (5.68)	2.2×10^{67} (3.2×10^{62}, 1.5×10^{72})	<.001

[a]Standard error
[b]Confidence interval
[c]Area deprivation index
[d]Box-Cox transformed

References

1. Silver ER, Truong HQ, Ostvar S, Hur C, Tatonetti NP. Association of Neighborhood Deprivation Index With Success in Cancer Care Crowdfunding. *JAMA Netw Open*. 2020;3(12):e2026946. doi:10.1001/jamanetworkopen.2020.26946
