# Supplementary material for: Impact of Medical Conditions and Area Deprivation on Fundraising Success in Online Crowdfunding: Cross-Sectional Study
Source: J Med Internet Res. 2025 Jul 29;27:e72475. doi: 10.2196/72475 (PMC12306843; doi:10.2196/72475)
Supplement: Checklist 1 [file jmir-v27-e72475-s003.docx]

STROBE Statement—Checklist of items that should be included in reports of ***cross-sectional studies***

|  | Item No | Recommendation | Relevant text from this manuscript |
| --- | --- | --- | --- |
| **Title and abstract** | 1 | (*a*) Indicate the study’s design with a commonly used term in the title or the abstract | “…: Cross-Sectional Study” |
|  |  | (*b*) Provide in the abstract an informative and balanced summary of what was done and what was found | See “Abstract” |
| Introduction | | |  |
| Background/rationale | 2 | Explain the scientific background and rationale for the investigation being reported | “Despite the breadth of previous work, most studies…” |
| Objectives | 3 | State specific objectives, including any prespecified hypotheses | “We hypothesized that campaigns would frequently mention…” |
| Methods | | |  |
| Study design | 4 | Present key elements of study design early in the paper | “We wrote a web scraping program to…” |
| Setting | 5 | Describe the setting, locations, and relevant dates, including periods of recruitment, exposure, follow-up, and data collection | “We wrote a web scraping program to…” |
| Participants | 6 | (*a*) Give the eligibility criteria, and the sources and methods of selection of participants | “Of the 99,943 crowdfunding campaigns…” |
| Variables | 7 | Clearly define all outcomes, exposures, predictors, potential confounders, and effect modifiers. Give diagnostic criteria, if applicable | “To estimate the impact of disease categories and ADI quartile on fundraising success…” |
| Data sources/ measurement | 8* | For each variable of interest, give sources of data and details of methods of assessment (measurement). Describe comparability of assessment methods if there is more than one group | “Two sensitivity analyses were conducted…” |
| Bias | 9 | Describe any efforts to address potential sources of bias | “A discussion on the possibility of bias in our sample is available in Texts S1- and S2 and Figure S1 in Multimedia Appendix 1.” |
| Study size | 10 | Explain how the study size was arrived at | “Per guidance from our institutional review board, we collected data from no more than five 5percent% of campaigns available on the GFM sitemap, resulting in a maximum sample size of 100,000.” |
| Quantitative variables | 11 | Explain how quantitative variables were handled in the analyses. If applicable, describe which groupings were chosen and why | “…we constructed a generalized linear model (GLM) with the amount of money raised as the dependent variable.”  “We suppose that the number of donations received is Poisson-distributed…” |
| Statistical methods | 12 | (*a*) Describe all statistical methods, including those used to control for confounding | See “Statistical Analysis” section for complete details |
|  |  | (*b*) Describe any methods used to examine subgroups and interactions | N/A |
|  |  | (*c*) Explain how missing data were addressed | “we excluded campaigns that were duplicated (n=6,981), had missing or unmappable zip codes (n=421)…”  “Two sensitivity analyses were conducted…” |
|  |  | (*d*) If applicable, describe analytical methods taking account of sampling strategy | “…to collect data from a random sample of medical crowdfunding campaigns…” |
|  |  | (*e*) Describe any sensitivity analyses | “Two sensitivity analyses were conducted…” |
| Results | | |  |
| Participants | 13* | (a) Report numbers of individuals at each stage of study—eg numbers potentially eligible, examined for eligibility, confirmed eligible, included in the study, completing follow-up, and analysed |  |
|  |  | (b) Give reasons for non-participation at each stage | N/A |
|  |  | (c) Consider use of a flow diagram | N/A |
| Descriptive data | 14* | (a) Give characteristics of study participants (eg demographic, clinical, social) and information on exposures and potential confounders | “The final sample contained 89,645 unique medical crowdfunding campaigns. Campaigns were created from 2010 to 2020, with only 1.2% (n=1094) of campaigns starting before 2014.”  See Table 1 |
|  |  | (b) Indicate number of participants with missing data for each variable of interest |  |
| Outcome data | 15* | Report numbers of outcome events or summary measures | N/A |
| Main results | 16 | (*a*) Give unadjusted estimates and, if applicable, confounder-adjusted estimates and their precision (eg, 95% confidence interval). Make clear which confounders were adjusted for and why they were included | See Figure 2 |
|  |  | (*b*) Report category boundaries when continuous variables were categorized | N/A |
|  |  | (*c*) If relevant, consider translating estimates of relative risk into absolute risk for a meaningful time period | N/A |
| Other analyses | 17 | Report other analyses done—eg analyses of subgroups and interactions, and sensitivity analyses | “Two sensitivity analyses were conducted…” |
| Discussion | | |  |
| Key results | 18 | Summarise key results with reference to study objectives | “In this analysis of online web-based medical crowdfunding campaigns, the success of campaigns was influenced by the specific diseases mentioned in the campaign description as well as the socioeconomic deprivation of the county in which the campaign was initially posted. In addition, differences in fundraising were more often explained by the number of donations received rather than the mean donation amount.” |
| Limitations | 19 | Discuss limitations of the study, taking into account sources of potential bias or imprecision. Discuss both direction and magnitude of any potential bias | See “Limitations” section for complete details |
| Interpretation | 20 | Give a cautious overall interpretation of results considering objectives, limitations, multiplicity of analyses, results from similar studies, and other relevant evidence | See paragraphs 2-8 in the “Discussion” section |
| Generalisability | 21 | Discuss the generalisability (external validity) of the study results | See paragraphs 2-8 in the “Discussion” section and “Limitations” section |
| Other information | | |  |
| Funding | 22 | Give the source of funding and the role of the funders for the present study and, if applicable, for the original study on which the present article is based | See “Acknowledgements” section |

*Give information separately for exposed and unexposed groups.

**Note:** An Explanation and Elaboration article discusses each checklist item and gives methodological background and published examples of transparent reporting. The STROBE checklist is best used in conjunction with this article (freely available on the Web sites of PLoS Medicine at http://www.plosmedicine.org/, Annals of Internal Medicine at http://www.annals.org/, and Epidemiology at http://www.epidem.com/). Information on the STROBE Initiative is available at www.strobe-statement.org.
